# Antimalarial Activity of Crude Extract and Solvent Fractions of Leaves of *Solanum nigrum L*. (*Solanaceae*) against *Plasmodium berghei* in Mice

**DOI:** 10.1155/2022/3426175

**Published:** 2022-07-18

**Authors:** Eshetie Melese Birru, Muluken Adela Alemu, Asegedech Tsegaw Weredekal, Assefa Belay Asrie, Mestayet Geta Mengistie

**Affiliations:** ^1^Department of Pharmacology, School of Pharmacy, College of Medicine and Health Sciences, University of Gondar, P.O. Box 196, Gondar, Ethiopia; ^2^Department of Pharmacy, College of Medicine and Health Sciences, Debre Tabor University, P.O. Box 272, Debre Tabor, Ethiopia

## Abstract

**Background:**

Current malaria treatment is associated with continued development of drug resistance. Thus, there is a need to develop safe and effective new treatments from different sources. *Solanum nigrum* L. (*Solanaceae*) is a plant used for the treatment of malaria in Ethiopian traditional medicine. This study was aimed at evaluating of antimalarial activity of the crude extract and fractions of *S. nigrum* L. (*Solanaceae*) leaves against *P. berghei* infection in mice.

**Method:**

Both prophylactic and suppressive models were used in evaluating antimalarial activity using the ANKA *Plasmodium* strain. In these models, male mice were randomly grouped into eleven groups (*n* = 5). Mice in group I were given 4% Tween-80, mice from group II up to X were given 100, 200, and 400 mg/kg of plant extract, and the last group (XI) was treated with chloroquine (25 mg/kg). Data were analyzed using one-way ANOVA followed by post hoc Tukey's multiple comparison test.

**Results:**

Crude extract of leaves of *S. nigrum* showed chemosuppression of 30.68 (*P* < 0.05), 42.42 (*P* < 0.01), and 50.75% (*P* < 0.001) at 100, 200, and 400 mg/kg doses of the extract, respectively. At doses of 100, 200, and 400 mg/kg, the chloroform fraction produced a chemosuppressive effect of 40.15% (*P* < 0.01), 53.78% (*P* < 0.001), and 65.15% (*P* < 0.001) and a chemoprophylactic effect of 42.7% (*P* < 0.05), 51.84% (*P* < 0.001), and 67.17% (*P* < 0.001) when compared with negative control. In the suppressive model, the ethyl acetate fraction demonstrated a mean chemosuppression of 56.81% (*P* < 0.001), 65.9% (*P* < 0.001), and 70.83% (*P* < 0.001). Similarly, in the prophylactic model, the fraction showed suppression of 42.70% (*P* < 0.05), 53.11% (*P* < 0.001), and 71.03% (*P* < 0.001) at 100, 200, and 400 mg/kg doses. On the acute oral toxicity test, the extracts were safe at 2 g/kg dose.

**Conclusion:**

*S. nigrum* L. has antimalarial activity and supports the traditional medical practice.

## 1. Introduction

Globally, a total of an estimated 405,000 deaths and 228 million new malaria cases occurred due to malaria in 2018. Among these malaria deaths and cases, more than 90% were in the African region. Morbidity and mortality due to malaria are very common in children [1]. Next to *P. falciparum, P. vivax* is the second main cause of morbidity and mortality in causing severe malaria and death. In the world, *P. vivax* accounts for around 9% of estimated malaria cases, although the proportion excluding the African countries is significantly higher (50%) [1, 2].

The drug of choice for treating malaria depends on various factors, including the severity of the infection, patient-related factors, parasite sensitivity, and the cost and availability of the drugs. Therapeutic goals include prophylaxis, treatment of the acute attack (clinical cure), and radical cure via elimination of dormant malaria parasites from the liver in the case of *P. ovale* and *P. vivax* malaria infection and to block malaria transmission [3]. Unfortunately, the malaria parasites have developed resistance to almost all of the known antimalarial drugs in different parts of the world [4, 5]. In response to this, the latest effective antimalarial drugs like artemisinin and its derivatives (artemether and arteether) are part of the WHO-recommended first-line antimalarial drugs [6, 7].

While artemisinin-based combination therapies (ACT) are recommended as first-line treatments for uncomplicated malaria, the emergence of the artemisinin-resistant parasite in countries (Bangladesh and Thailand) makes the choice of these drugs difficult [8] and abets finding new treatment regimens [9]. Hence, developing new medicinal agents from plants as used in history is continued to serve as the basis for many pharmaceuticals used today. In Ethiopian traditional medicinal practice, many people use herbal antimalarial preparations as they are easily accessible and affordable. However, scientific evidence on the safety and efficacy of those herbal medicines and isolation of active principles is very minimal [10, 11].

*Solanum nigrum* is a medicinal plant belonging to the family *Solanaceae*, genus of *Solanum* (nightshade), and species of *S. nigrum* L. Its common names are awut (*Amharic*) and black nightshade (*English*) [11–13]. There are two varieties of *S. nigrum*, i.e., black colour fruit and reddish-brown colour fruit. Both of the varieties have been used traditionally to treat various ailments such as pain, inflammation and fever [14, 15], malaria [16], and enteric diseases [17]. *S. nigrum* possess several pharmacological activities like antitumorigenic and antioxidant [18], anti-inflammatory [15], hepatoprotective [19], diuretic [15, 20], antipyretic [15, 20], antibacterial [17], antifungal [21], and cytotoxicity, anti-convulsant, and antiulcerogenic [22, 23]. In addition to the claimed traditional use, the 80% hydromethanolic fruit extract of the plant has promising antimalarial activity, *in vitro* (IC_50_ = 10.29 *μ*g/ml) and *in vivo* with a percentage parasitemia inhibition of 60.68% (*P* < 0.05). Additionally, in the *in vitro* test, the 50% cytotoxic concentration (CC_50_) of the fruit extract of the plant was 66.3 ug/ml on the Raji cell line (B lymphocyte cell line), showing that the plant is potentially toxic at much higher doses [24]. The previous studies on the fruit of the plant did not address malarial prophylactic effects and acute oral toxicity as well as the antimalarial effect of different solvent fractions [16, 24, 25]. To fill these gaps, the current study is pointed at evaluating *in vivo* antiplasmodial activity of crude extract and solvent fractions of *S. nigrum* L. leaves.

## 2. Methods

### 2.1. Collection and Preparation of the Plant Materials

After obtaining permission from the owner of the farmland, the fresh leaves of *S. nigrum* were collected in June 2016, around Gondar, Zegie Peninsula, South West of Lake Tana, Northwest Ethiopia. The use of the plant in the present study complies with the IUCN (International Union for Conservation of Nature) Policy Statement on Research Involving Species at Risk of Extinction and the Convention on the Trade in Endangered Species of Wild Fauna and Flora. The plant was identified and authenticated by Mr. Abiyu Mola (Assistant Professor of Botanical Science) in the Department of Biology, College of Natural and Computational Science, University of Gondar, and a voucher specimen (with a voucher number of MG01) was deposited at National Herbarium, Addis Ababa University, Ethiopia. The leaves of *S. nigrum* were cleaned and air-dried in a shaded area at room temperature. Then after being coarsely powdered, it was stored in a plastic container and kept at room temperature until extraction.

### 2.2. Extraction of the Plant Materials

The powdered samples of leaves of *S. nigrum* were weighed using a Wensar analytical balance (Swastik Systems and Services, India). Then 800-gram powder was extracted by cold maceration with 80% hydromethanolic solvent (80 ml of methanol added with 20 ml of distilled water) for three days. After 72 h, the mixture was filtered using Whatman filter paper number 1. After that, the residue was macerated with ethyl acetate for 72 h and filtered once more. Finally, chloroform was added to the residue and was macerated for another 72 h, followed by filtration. The same process of extraction was repeated using the residue, ethyl acetate, and chloroform [26]. The combined filtrates were dried by oven at a temperature below 40°C and then weighed to determine the percentage yield. Finally, it was transferred to a vial and kept in a refrigerator until use.

### 2.3. Phytochemistry Screening

Preliminary phytochemical screening of the leaves crude extract was performed as per the standard screening methods [27, 28].

### 2.4. Acute Toxicity Test

The crude hydromethanolic extract and solvent fractions of *S. nigrum* were tested in noninfected female Swiss albino mice aged 6–8 weeks and weighing 20–30 g. For the crude hydromethanolic extract and solvent fractions, 15 mice were divided into three groups randomly (5 mice in each group). After 3 hrs of food starvation, excluding water, the mice received a single oral dose of the extract. The mice in groups I, II, and III were given 2000 mg/kg of the crude hydromethanolic extract, chloroform, and ethyl acetate fraction, respectively, dissolved in 10 ml/kg of the vehicle. The mice in the control group were given 10 ml/kg of the vehicle (4% Tween 80 in distilled water) used for dissolution of the extract. The mice were observed for 1 h continuously and every 4 h for 24 hrs and daily for 14 days for any manifestation of toxicity [29].

### 2.5. Experimental Animals

One hundred ten Swiss albino male mice, weighing 20 to 30 g and 6 to 8 weeks old inbred at the Animal House of the Department of Pharmacology, University of Gondar, were used. They were maintained in plastic cages, and the beddings were made of softwood chips and shavings. Animals were exposed to a 12 : 12 dark-to-light cycle and had free access to pelleted food and drinking water. All mice were acclimatized to the laboratory room 7 days before the start of the actual experiment [30].

### 2.6. Parasites

*Plasmodium berghei* (ANKA strain), which was chloroquine-sensitive, was used in this experiment. The donor *P. berghei*-infected mice were taken from Aklilu Lemma Institute of Pathobiology, Addis Ababa University, Ethiopia. Mice were first anesthetized by ketamine and then sacrificed using cervical dislocation, and the parasites were transferred from mice to mice through the passage of blood from infected donor mice to naive ones through intraperitoneal (IP) route weekly [31].

### 2.7. Drugs and Reagents

Normal saline (Addis Pharmaceuticals Factory, Ethiopia), methanol (Okhla Industrial, India), Giemsa (Sciencelab, USA), chloroquine phosphate 250 mg tablet (Addis Pharmaceuticals Factory, Ethiopia), ketamine (Rotexmedica, Germany), and trisodium citrate (Deluxe Scientific Surgico, India) were used. All these reagents were obtained from certified suppliers and were of analytical grade.

### 2.8. Study Design and Sampling Method

An experimental study design was employed and a simple random sampling technique was used for the assignment and grouping of mice.

### 2.9. Inoculum Preparation

After anaesthetizing with ketamine, a cardiac puncture was used to collect blood from *P. berghei*-infected donor mouse with a rising parasitemia of 30 to 35% into a test tube having 0.5% trisodium citrate. Then the blood was diluted with 0.9% normal saline to give 2 × 10^7^ IRBCs (Infected Red Blood Cells) in an injection volume of 0.2 ml. The amount of normal saline used in the dilution was determined by the level of parasitemia (%) of the infected donor mice. Each mouse was then infected by injecting diluted blood (0.2 ml) intraperitoneally, which contained 2 × 10^7^ IRBCs and produced a steadily rising infection in the recipient mice [31].

### 2.10. Grouping and Dosing of Animals

For both models (suppressive and prophylactic), fifty-five mice were grouped randomly into eleven groups of five mice each. Mice in group I were treated with the vehicle (4% Tween 80 in distilled water, 10 ml/kg, served as negative control), mice in groups II, III, and IV were treated with 100, 200, and 400 mg/kg of the crude hydromethanolic extract, groups V, VI, and VII were treated with 100, 200, and 400 mg/kg of the chloroform extract, and groups VIII, IX, and X were treated with 100, 200, and 400 mg/kg of the ethyl acetate extract, respectively. The doses of the extracts were determined as 100, 200, and 400 mg/kg based on acute toxicity test results and OECD (Organization for Economic Cooperation and Development) guideline recommendations. That is, one-tenth of 2000 mg/kg of the dose used in the acute oral toxicity test is used to determine the middle dose, and one-half of and 2 times the middle dose were used to determine the lowest and highest doses, respectively. The last group (XI) of mice were given the standard drug (chloroquine, 25 mg/kg), which served as positive control.

Three testing dose levels (100, 200, and 400 mg/kg) of the crude extract and solvent fractions were selected to evaluate antimalarial activity. As people in traditional medicine use the plant material for the prevention and treatment of malaria through oral administration [16, 25], each treatment dose was administered through an oral route using oral gavage to ensure safe ingestion of the preparations.

### 2.11. Four-Day Suppressive Antimalarial Test

The test provides preclinical information on the presence or absence of antimalarial activity of the plant extracts [31, 32]. The method described by Fidock et al. was used [31]. After inoculating the standard parasite (2 × 10^7^*P. berghei* IRBCs, 0.2 ml), fifty-five mice were randomly grouped into eleven groups as mentioned above. Three hrs after infection, treatment was started and then continued for three consecutive days, i.e., from D0 to D3, with respective preparations as described above. Finally, thin blood films were made on a microscope slide from the tail of each mouse to determine the level of parasitemia on the 5^th^ day (D4).

### 2.12. Prophylactic Antimalarial Test

Considering the promising antimalarial activity of the plant material in the 4-day suppressive assays, further evaluation with other tests was found justifiable. Hence, the evaluation of the prophylactic potential of the extract was done according to Fidock et al. [31]. Fifty-five mice were randomly grouped into eleven groups, as mentioned above accordingly, and then treated for four consecutive days (D0 to D3). On the fifth day (D4), each mouse was exposed to a standard inoculum (2 × 10^7^*P. berghei* IRBCs, 0.2 ml) intraperitoneally. After 72 hrs, thin blood smears were prepared using microscopic slides from the tail tip of each mouse.

### 2.13. Peripheral Blood Smear Preparation

Thin smears of blood were made from the tail of each mouse on the fifth (D4) and eighth (D7) days for both suppressive and prophylactic models, respectively. Slides were dried, fixed, and stained at pH 7.2 for 10 minutes using 10% Giemsa and were washed gently using distilled water and at the end air-dried at room temperature.

### 2.14. Parasitemia Determination

Finally, the slides were examined under a microscope with an oil immersion objective (×100 magnification power) by taking an average of six fields. Percentage parasitemia was calculated by dividing the number of infected RBC by the total RBC from Giemsa stained thin blood films and multiplying it by 100. The percent suppression of parasitemia was calculated for each dose level by dividing the percent parasitemia difference between the negative control and treated group by the percent parasitemia in the negative control group multiplied by 100, as shown in the following formula [31]:

(1) $$\begin{array}{c} \text{\% parasitemia}=\frac{\text{number of parasitized RBC }}{\text{total number of RBC}}*100 \end{array}$$

(2) $$\begin{array}{c} \text{\% suppression}=\frac{\left(\text{parasitemia of negative control}-\text{mean parasitemia of treated group}\right)}{\text{mean parasitemia of negative contol }}*100. \end{array}$$

### 2.15. Determination of Mean Survival Time

Mean survival time (MST) is the other common parameter used to evaluate the efficacy of antimalarial plant extract [32]. An extract is considered active if it demonstrates a longer survival time of an infected experimental animal as compared to nontreated mice. In both models, each mouse was observed from the date of standard inoculation (D0) throughout the whole follow-up period, and the mean survival time was calculated for each group using the following formula:

(3) $$\begin{array}{c} \text{MST}=\frac{\text{sum of survival time for all mice in a group }\left(\text{in days}\right)}{\text{total numbers of mice in that group}}. \end{array}$$

### 2.16. Determination of Body Weight and Temperature

More often, the changes in body weight and temperature are used as a parameter to evaluate the efficacy of extracts against malaria infection. In a four-day suppressive antimalarial test, the body weight and temperature were taken on day zero and day four. In the case of the prophylactic test, the body weight and rectal temperature of each mouse were taken before treatment (D1) and on day seven (D7).

### 2.17. Determination of Packed Cell Volume

Determination of packed cell volume (PCV) is helpful in assessing the effectiveness of the extract in preventing red blood cell degradation associated with the overgrowing *Plasmodium* parasite. Blood was collected from the tail of each mouse in heparinized microhematocrit capillary tubes by filling three-quarters of its volume and sealed with clay. The sealed tubes were allowed to centrifuge in microhematocrit centrifuge with a speed of 12,000 rates per minute. After five minutes, the sealed tubes were removed from microhematocrit centrifuge and the percentage of PCV was read using a calibrated microhematocrit reader. In both the suppressive and prophylactic tests, PCV was determined using the following formula:

(4) $$\begin{array}{c} \text{PCV}=\frac{\text{volume of erythrocytes in a given volume of blood }}{\text{total blood volume }}. \end{array}$$

### 2.18. Data Analysis

The data of the study were expressed as mean ± SEM (standard error of mean) for each group of experiments. Data on the levels of parasitemia, variations in body weight, survival times, packed cell volume, and body temperature were analyzed using Windows SPSS version 20. The differences between means of parasitemia, survival date, PCV, body weight, and temperature were compared using one-way ANOVA followed by Turkey's HSD multiple comparison test. The *P* values < 0.05 were regarded as statistically significant.

### 2.19. Data Quality Control

Data quality was maintained and ensured by randomization of animals and treatment assignments. Codes were used for all microscopic slides and we used a blinding technique for the reader to minimize bias.

### 2.20. Ethical Consideration

During the experiment, mice were cared and handled according to the internationally accepted laboratory animals' care, use, and welfare guidelines [30]. Each mouse was sacrificed using cervical dislocation after completing the study. Ethical issues and the study protocol were approved by the Institutional Review Board of the University of Gondar (v/p/rcs/237/2017) and the study complied with the ARRIVE guideline.

## 3. Results

### 3.1. Yields of the Extracts

After successive extraction of 800 g of the powder by 80% hydromethanol, ethyl acetate, and chloroform, the yields were 120 g (15%), 10 g (1.25%), and 8 g (1%)

### 3.2. Phytochemical Screening

Preliminary phytochemical screening of the leaves crude hydromethanolic extract of *S. nigrum* revealed the presence of phenols, flavonoids, glycosides, alkaloids, saponins, steroids, tannins, and terpenoids (Table 1).

### 3.3. Acute Oral Toxicity Test

As observed from the *in vivo* acute toxicity study result of the extracts, there were no gross physical and behavioral changes such as sleep, rigidity, depression, diarrhoea, hair erection, and abnormal secretion for 24 h. All mice survived until the end of the observation period (two weeks).

### 3.4. Four-Day Suppressive Antimalarial Activity

After a four-day suppressive test, the crude hydromethanolic extract and solvent fractions of leaves of *S. nigrum* showed significant dose-dependent anti-plasmodial activity against chloroquine-sensitive *P. berghei*-infected Swiss albino mice tested doses as compared to the negative control (4% Tween 80). The crude hydromethanolic extract has shown % suppression of 30.68% at 100 mg/kg, 42.42% at 200 mg/kg and 50.75% at 400 mg/kg and also the chloroform fraction suppressed parasitemia by 40.15% at 100 mg/kg, 53.78% at 200 mg/kg and 65.15% at 400 mg/kg. Similarly, ethyl acetate fraction has shown % suppression of 56.81, 65.9, and 70.83 at doses of 100 mg/kg, 200 mg/kg, and 400 mg/kg, respectively (Table 2).

#### 3.4.1. Determination of Mean Survival Time

Except for 400 mg/kg dose of ethyl acetate fraction (*P* < 0.05), none of the doses of the crude hydromethanolic extract and solvent fractions of *S. nigrum* significantly prolonged survival days of *P. berghei*-infected mice as compared to the vehicle-treated control group as indicated in Table 2.

#### 3.4.2. Effect on Body Weight

In this test, neither the crude extract nor the solvent fraction doses prevented loss of body weight significantly as compared with negative control, as shown in Table 3.

#### 3.4.3. Effect on Packed Cell Volume (PCV) and Body Temperature

From all the treatment groups, only the ethyl acetate fraction at the dose of 400 mg/kg significantly protected the mice from PCV reduction as compared with the negative control. Neither the doses of the crude hydromethanolic extract nor the solvent fractions of *S. nigrum* significantly prevented the reduction in body temperature of infected mice as compared with the negative control (Table 4).

### 3.5. Chemoprophylactic Antimalarial Activity of the Extract

The crude hydromethanolic extract and solvent fractions of *S. nigrum* leaves also showed chemoprophylactic activity against *Plasmodium berghei* infection in mice with crude extract 30.73%, 42.91%, and 51.70%, chloroform fraction 42.70%, 51.84% and 67.17% and those of ethyl acetate fractions were 42.70%, 53.11%, and 71.03% at 100, 200, and 400 mg/kg doses, respectively. The group that received 400 mg/kg of the ethyl acetate fraction exhibited maximal suppression (71.03%) of parasitemia compared to the other groups (Table 5). All plant material treatments suppressed the level of parasitemia significantly (*p* < 0.05, *p* < 0.01, *p* < 0.001) as compared to the vehicle-treated mice dose-dependently.

#### 3.5.1. Effect of the Extract on Mean Survival Time

In the prophylactic test, 400 mg/kg chloroform and ethyl acetate fraction treated groups lived for a statistically significant (*P* < 0.05) longer period as compared to distilled water treated group.

#### 3.5.2. Effect on Body Weight

All the extract doses did not prevent body weight loss significantly (Table 6) as compared to the negative control, except the 400 mg/kg dose of ethyl acetate fraction (*P* < 0.05).

#### 3.5.3. Effect on Packed Cell Volume (PCV) and Body Temperature

Except for the ethyl acetate fraction at 200 mg/kg and 400 mg/kg doses (*P* < 0.05), the crude extract and the solvent fraction doses did not prevent the hemolysis of red blood cells of the mice significantly as compared to the vehicle-treated group. Within plant material treatment groups, the analysis also revealed no apparent difference in the mean value of the PCV of mice, as depicted in Table 7. Similarly, none of the test groups demonstrated significant prevention of body temperature reduction when compared to each other and the negative control. However, the ethyl acetate fraction at 400 mg/kg averted temperature loss significantly (*P* < 0.05) as compared to the distilled water treated group.

## 4. Discussion

The majority of African countries, including Ethiopia, rely on herbal products for their healthcare needs. With regard to Ethiopia, the country is generally poor and over two-thirds of the population live in areas where malaria is a high risk. Moreover, due to the accessibility of traditional medicine and long-lasting cultural experience not only for malaria but also for various health ailments, plants are used as therapeutic agents. Even though hundreds of medicinal plants are in used in Ethiopia daily, the efficacy and safety issues are not well understood [33].

Acute toxicity test results of the crude hydromethanolic extract of the leaves of *S. nigrum* showed no sign of toxicity in all exposed mice. From this, it is possible to conclude that the LD_50_ of the extract is beyond 2000 mg/kg [OECD] and as per WHO hazard classification, the plant material is designated as “unlikely to be hazardous” [34]. In the present study, to extract polar and moderately polar compounds, 80% hydromethanol, a universal solvent, and a cold maceration technique were used, which is preferable for the efficient recovery of plant constituents.

In this study, both the 4-day suppressive and prophylactic tests of the crude extract and the solvent fraction of the leaves of *S. nigrum* demonstrated significant inhibition of parasitemia level at three dose levels. In evaluating antimalarial activity, determining percent parasitemia suppression and survival time are the most reliable parameters [35]. A compound is considered active when percent suppression in parasitemia is 30% or more [36, 37], and therefore, the leaves extracts of *S. nigrum* possess a promising antimalarial activity. The highest percentage of parasitemia suppression in both models was exhibited by the ethyl acetate fraction at 400 mg/kg (70.83% in the 4-day suppressive test and 71.03% in the prophylactic test) (*P*  < 0.001), followed by the chloroform fraction at 400 mg/kg (65.15% in the 4-day suppressive and 67.13% in the prophylactic test) (*P* < 0.001). Hence, in both models, the antiplasmodial activity of the plant is observed to be dose-dependent. Dose-dependent antimalarial activity is also reported for other plants, including *Euphorbia abyssinica*, being 66.87, 84.94, and 93.68% at 200, 400, and 600 mg/kg doses, respectively. Similarly, *Indigofera spicata* showed parasitemia suppression of 34.93 and 53.42% at 400 and 600 mg/kg doses [38, 39], and the antimalarial activity of *S. nigrum* is also supported by other related species such as *Solanum surattense* (IC_50_ < 50 ug/ml) [40] and *Solanum tuberosum* [41].

Both the crude extract and the solvent fractions of *S. nigrum* showed considerable antimalarial properties. This might suggest that the antimalarial activity of the plant may arise from a single or combination of active phytochemical ingredients. The difference in percent parasitemia suppression between the fractions might be due to the quantitative and/or qualitative differences among the bioactive constituents in each fraction. As stated above, the highest activity was from the ethyl acetate fraction, justifying that the solvent localized noteworthy bioactive secondary metabolites in kind and concentration. Through the isolation of the biologically active agents from the most active fraction, a new antimalarial drug could be identified for future development.

Mean survival time as an additional parameter in evaluating antimalarial activity indicates that a test substance is active against malaria if it can prolong the MST of the infected animals as compared to the negative control [31, 42]. Mice treated with 400 mg/kg ethyl acetate fraction in the 4-suppressive test and mice treated with 400 mg/kg chloroform and the ethyl acetate fractions in the prophylactic test survived for a longer period (*P* < 0.05) than vehicle-treated mice. This might be because of the inherent antiplasmodial activity of the extract. However, when compared with the positive control, the survival times of mice treated with the extract were shorter, which might be due to uninvestigated pharmacokinetic limitations such as rapid elimination of the extract. These results were in line with the studies done on the antimalarial activity of plants such as *Dodonaea angustifolia* [43] and *Nigella sativa* [44].

Haematological abnormalities like anaemia, weight loss, and temperature reductions are common characteristics of *P. berghei*-infected mice [45, 46]. Plants with antimalarial activity are expected to prevent such abnormalities in infected mice resulting from invasion of the parasite on RBC and the associated oxidative stress and decrease in the metabolic rate before death, which as a result causes a drop in internal body temperature [46, 47]. However, except for the ethyl acetate fraction, the crude extract of *S. nigrum* in both models did not prevent the loss in PCV, which might be due to the presence of saponins, affecting membrane permeability and leading to hemolysis [45, 48]. Likewise, in the other two evaluation parameters, body weight and temperature, in the 4-day suppressive test, the extract did not prevent body weight and temperature loss, while in the prophylactic test, the highest dose of the ethyl acetate fraction prevented significantly (*P* < 0.05), inferring its high parasitemia suppression. Loss of body weight prevention is a characteristic of the extracts' antimalarial efficacy since *Plasmodium berghei* infection in mice results in metabolic disturbance, appetite loss, and hypoglycemic effect [49].

Dahro et al.'s antimalarial activity classification stated that *in vivo* antiplasmodial activity can be classified as moderate, good, and very good if an extract displayed percentage parasitemia suppression equal to or greater than 50% at doses of 500, 250, and 100 mg/kg body weight per day, respectively [47, 49]. Based on this classification, in both models, the crude hydromethanolic extract and chloroform fraction of *S. nigrum* showed moderate and good antiplasmodial activities, respectively. Similarly, the ethyl acetate fraction showed good and very good antiplasmodial activities in the four-day suppressive and prophylactic tests, respectively.

As per the preliminary phytochemical screening result, *S. nigrum* has various secondary plant metabolites such as alkaloids, phenols, flavonoids, steroids, tannins, terpenoids, and glycosides. This has a strong similarity with the previous phytochemical report of the plant and the related species [25]. A huge number of phytochemicals show effective antimalarial activity belonging to the class of alkaloids, terpenes, flavonoids, sesquiterpenes, and other related compounds. Therefore, the demonstrated antimalarial activity of *S. nigrum* might be attributed to the phytochemicals present in the extract, which may work individually and/or synergistically with plenty of possible parasite suppressive mechanisms. Furthermore, the antimalarial activities of the plant may not be only by directly attacking the infecting *Plasmodium* parasite but also indirectly enhancing the defense mechanisms of the host [50] and ameliorating the oxidative stress via free radical scavenging activity [18–20]. Overall, considering all the aforementioned points, the extract of *S. nigrum* is reasonably a potential antimalarial agent, justifying the claimed use of the plant for malaria control and also the *in vitro* report.

## 5. Conclusion

From this study, it can be concluded that the leaves of the crude hydromethanolic extract and solvent fractions of *Solanum nigrum* L. showed good and very good chemosuppressive and chemoprophylactic activities, respectively, supporting the traditional use. Additionally, researchers may use the plant since it may serve as a potential source for new antimalarial compound(s) that may have a substantial contribution to malaria control and elimination.

## Figures and Tables

**Table 1:** Preliminary phytochemical screening of crude hydromethanolic extract and solvent fractions of the leaves of *S. nigrum*.

	Phenol	Flavonoid	Glycoside	Alkaloid	Saponin	Steroid	Tannin	Anthraquinone	Terpenoid
Crude hydromethanolic extract	+	+	+	+	+	+	+	−	+
Ethyl acetate fraction	+	+	+	+	+	+	+	−	+
Chloroform fraction	+	+	+	+	−	+	+	−	−

+ = presence; − = absence.

**Table 2:** Effect of crude hydromethanolic extract and solvent fractions of the leaf of *S. nigrum* on parasitemia level, % suppression, and mean survival time.

Treatments	Dose	% parasitaemia	% suppression	MST (day)
4% Tween 80	10 ml/kg	26.40 ± 1.36	0.00	6.40 ± 0.24
Chloroquine	25 mg/kg	0.00 ± 0.00	100.00	28.40 ± 0.50^a3^
Crude hydromethanolic extract	100 mg/kg	18.30 ± 0.86	30.68^a2b3^	7.80 ± 0.37^b3^
	200 mg/kg	15.20 ± 0.86	42.42^a3b3^	8.00 ± 0.44^b3^
	400 mg/kg	13.00 ± 0.70	50.75^a3b3^	8.40 ± 0.40^b3^
Chloroform fraction	100 mg/kg	15.80 ± 1.24	40.15^a3b3^	7.60 ± 0.40^b3^
	200 mg/kg	12.20 ± 1.32	53.78^a3b3^	7.80 ± 0.37^b3^
	400 mg/kg	9.20 ± 1.03	65.15^a3b3^	9.00 ± 0.31^b3^
Ethyl acetate fraction	100 mg/kg	11.40 ± 2.25	56.81^a3b3^	8.20 ± 0.37^b3^
	200 mg/kg	9.00 ± 0.65	65.9^a3b3^	8.80 ± 0.37^b3^
	400 mg/kg	7.70 ± 1.15	70.83^a3b3^	10.90 ± 0.40^a1b3^

Values are expressed as mean ± SEM; *n* = 5; a = as compared to the negative control; b = as compared to the positive control; 1 = *P* < 0.05; 2 = *P* < 0.01; 3 = *P* < 0.001; MST = mean survival time.

**Table 3:** Effect of crude hydromethanolic extract and solvent fractions of the leaf of *S. nigrum* L. on body weight of *P. berghei*-infected mice in a four-day suppressive test.

Treatments	Dose	Body weight (g)		
		D0	D4	W
4% Tween 80	10 ml/kg	26.32 ± 0.35	24.64 ± 0.42	−6.38
Chloroquine	25 mg/kg	26.60 ± 0.44	26.82 ± 0.26	0.83^a1^
Crude hydromethanolic extract	100 mg/kg	26.86 ± 0.86	25.48 ± 0.72	−5.14^b1^
	200 mg/kg	25.52 ± 0.79	24.36 ± 0.79	−4.54^b1^
	400 mg/kg	27.36 ± 0.82	26.60 ± 1.03	−2.78^b2^
Chloroform fraction	100 mg/kg	25.32 ± 0.90	23.87 ± 0.71	−5.73^b1^
	200 mg/kg	25.72 ± 0.79	24.54 ± 0.75	−4.59^b1^
	400 mg/kg	25.88 ± 0.85	25.12 ± 0.60	−2.94^b2^
Ethyl acetate fraction	100 mg/kg	24.74 ± 0.66	23.44 ± 0.75	−5.25^b1^
	200 mg/kg	25.96 ± 0.90	24.83 ± 0.66	−4.35^b1^
	400 mg/kg	23.46 ± 0.29	23.98 ± 0.52	−2.21^b2^

Values are expressed as mean ± SEM; *n* = 5. a = as compared to the negative control (4% tween- 80 as a vehicle) 10 ml/kg; b = as compared to the positive control (chloroquine 25 mg/kg); 3 = *P* < 0.05; 2 = *P* < 0.01; 1 = *P* < 0.001.

**Table 4:** Effect of crude hydromethanolic extract and solvent fractions of the leaf of *S. nigrum* L. on body temperature and packed cell volume of *P. berghei*-infected mice in the four-day suppressive test.

Treatments	Dose	% packed cell volume			Temperature (°C)		
		D0	D4	PCV	D0	D4	T
4% Tween 80	10 ml/kg	55.14 ± 1.33	49.81 ± 1.76	−9.67	36.40 ± 0.15	34.82 ± 0.12	−4.34
Chloroquine	25 mg/kg	55.14 ± 1.94	55.88 ± 0.32^a1^	1.34^a3^	36.31 ± 0.07	36.38 ± 0.25	0.19^a1^
Crude hydromethanolic extract	100 mg/kg	56.04 ± 3.96	52.46 ± 2.25^b1^	−6.39^b2^	36.30 ± 0.54	35.04 ± 0.59	−3.47
	200 mg/kg	52.40 ± 0.51	49.40 ± 0.51	−5.73^b2^	36.22 ± 0.39	35.12 ± 0.68	−3.04
	400 mg/kg	52.60 ± 0.51	50.00 ± 0.45	−4.94^b2^	35.88 ± 0.28	35.01 ± 0.24	−2.42
Chloroform fraction	100 mg/kg	55.80 ± 1.88	52.72 ± 2.06^b1^	−5.52^b2^	36.46 ± 0.42	35.03 ± 0.39	−3.92
	200 mg/kg	55.62 ± 1.28	53.34 ± 2.46	−4.10^b1^	35.69 ± 0.35	34.47 ± 0.24	−3.42
	400 mg/kg	56.00 ± 2.78	54.20 ± 3.13	−3.21^b1^	35.96 ± 0.36	35.04 ± 0.30	−2.56
Ethyl acetate fraction	100 mg/kg	55.28 ± 3.40	53.18 ± 2.53	−3.80^b1^	36.40 ± 0.63	35.22 ± 0.68	−3.24
	200 mg/kg	55.84 ± 1.07	54.02 ± 3.04	−3.26^b1^	36.24 ± 0.76	35.42 ± 0.67	−2.26
	400 mg/kg	54.24 ± 2.47	53.20 ± 2.27^a1^	−1.92^a1^	35.84 ± 0.47	35.56 ± 0.69	−0.78

Values are expressed as mean ± SEM; *n* = 5. a = as compared to the negative control (4% tween- 80 as a vehicle) 10 ml/kg; b = as compared to the positive control (chloroquine 25 mg/kg); 3 = *p* < 0.05; 2 = *p* < 0.01; 1 = *p* < 0.001

**Table 5:** Parasitemia level, % suppression, and mean survival time of crude extract and solvent fractions of the leaf of *S. nigrum* of *P. berghei*-infected mice in the prophylactic test.

Groups	Dose	% parasitemia	% suppression	MST (day)
4%Tween 80	10 ml/kg	28.24 ± 1.20	0	6.80 ± 0.37
Chloroquine	25 mg/kg	0 ± 0.00	100^a3^	28.80 ± 0.58
Crude hydromethanolic extract	100 mg/kg	19.56 ± 0.42	30.73^a1b3^	7.4000 ± 0.50^b3^
	200 mg/kg	16.12 ± 1.26	42.91^a2b3^	8.00 ± 0.70^b3^
	400 mg/kg	13.64 ± 0.34	51.7^a3b3^	8.60 ± 0.51^b3^
Chloroform fraction	100 mg/kg	16.18 ± 0.71	42.7^a2b3^	8.40 ± 0.51^b3^
	200 mg/kg	13.6 ± 0.79	51.84^a3b3^	9.00 ± 0.70^b3^
	400 mg/kg	9.28 ± 0.62	67.13^a3b3^	10.60 ± 0.51^a1b3^
Ethyl acetate fraction	100 mg/kg	16.18 ± 1.43	42.70^a2b3^	9.00 ± 0.71^b3^
	200 mg/kg	13.24 ± 0.51	53.11^a3b3^	9.60 ± 0.51^b3^
	400 mg/kg	8.18 ± 0.73	71.03^a3b1^	11.00 ± 0.71^a2b1^

Values are expressed as mean ± SEM; *n* = 5. a = as compared to the negative control; b = as compared to the positive control, 1 = *P* < 0.05; 2 = *P* < 0.01; 3 = *P* < 0.001.

**Table 6:** Effect of crude hydromethanolic extract and solvent fractions of the leaf of *S. nigrum* L. on body weight of *P. berghei*-infected mice in the prophylactic test.

Treatments	Dose	Body weight (g)		
		D3	D7	% W
4% Tween 80	10 ml/kg	25.10 ± .71	21.92 ± 0.54	−12.67
Chloroquine	25 mg/kg	24.52 ± 0.68	24.96 ± 0.50	1.79^a2^
Crude hydro-methanolic extract	100 mg/kg	23.96 ± .50	21.66 ± 0.66	−9.60^b1^
	200 mg/kg	26.78 ± 0.53	24.98 ± 0.45	−6.72
	400 mg/kg	25.20 ± 0.57	24.12 ± 0.75	−4.29
Chloroform fraction	100 mg/kg	26.79 ± 0.77	24.41 ± 1.37	−8.88
	200 mg/kg	23.87 ± 0.95	22.04 ± 0.93	−7.67
	400 mg/kg	26.22 ± 0.55	25.16 ± 0.32	−4.02
Ethyl acetate fraction	100 mg/kg	23.96 ± 0.65	22.05 ± 0.72	−7.97
	200 mg/kg	25.44 ± 0.93	23.82 ± 0.67	−6.37
	400 mg/kg	23.84 ± 1.65	23.02 ± 0.62	−3.44^a1^

Values are expressed as mean ± SEM; *n* = 5. a = as compared to the negative control (4% tween- 80 as a vehicle) 10 ml/kg; b = as compared to the positive control (chloroquine 25 mg/kg); 3 = *P* < 0.05; 2 = *P* < 0.01; 1 = *P* < 0.001.

**Table 7:** Effect of crude extract and solvent fractions of the leaf of *S. nigrum* L. on body temperature and packed cell volume of *P. berghei*-infected mice in the prophylactic test.

Treatments	Dose	% packed cell volume			Temperature (°C)		
		D3	D7	PCV	D3	D7	% Change
4% Tween 80	10 ml/kg	53.14 ± 2.43	47.08 ± 2.55	−11.40	35.86 ± 0.48	34.02 ± 0.51	−5.13
Chloroquine	25 mg/kg	54.46 ± .92	55.40 ± 1.16	1.73^a1^	36.44 ± 0.36	36.94 ± 0.25	1.37^a2^
Crude extract	100 mg/kg	52.80 ± 1.65	50.15 ± 3.21	−5.02^b1^	35.74 ± 0.35	34.26 ± 0.40	−4.14
	200 mg/kg	53.46 ± 2.10	51.20 ± 1.63	−4.23	36.62 ± 0.45	35.78 ± 0.41	−2.29
	400 mg/kg	52.92 ± .73	51.04 ± 1.51	−3.56	36.77 ± 0.30	36.02 ± 0.27	−2.04
Chloroform fraction	100 mg/kg	52.38 ± 1.46	49.74 ± 2.28	−5.04^b1^	35.56 ± 0.40	34.22 ± 0.32	−3.67
	200 mg/kg	52.60 ± 2.87	50.42 ± 1.56	−4.14	36.61 ± 0.33	35.39 ± 0.35	−3.33
	400 mg/kg	53.82 ± 1.06	51.60 ± 2.015	−4.12	35.95 ± 0.36	35.18 ± 0.28	−2.14
Ethyl acetate fraction	100 mg/kg	53.34 ± 1.77	50.22 ± 2.33	−5.85^b1^	35.88 ± 0.80	34.26 ± 0.81	−4.52
	200 mg/kg	52.91 ± 0.48	51.11 ± 1.93	−3.40^a1^	36.14 ± 0.47	34.92 ± 0.24	−3.36
	400 mg/kg	52.16 ± 1.74	50.45 ± 1.49	−3.28^a1^	36.22 ± 0.38	35.54 ± 0.34	−1.88^a1^

Values are expressed as mean ± SEM; *n* = 5. a = as compared to the negative control (4% Tween 80 as a vehicle) 10 ml/kg; b = as compared to the positive control (chloroquine 25 mg/kg); 3 = *P* < 0.05; 2 = *P* < 0.01; 1 = *P* < 0.001.

## Data Availability

The datasets used and/or analyzed during the current study are available from the corresponding author on reasonable request.

## Abbreviations

ANKA: ANtwerpen and KAtanga
ANOVA: Analysis of variance
IP: Intraperitoneal
IRBC: Infected red blood cells
IUCN: International Union for Conservation of Nature
MST: Mean survival time
NS: Normal saline
OECD: Organization for Economic Cooperation and Development
PCV: Packed cell volume
WHO: World Health Organization.
